# Digital Media Use and Adolescents' Mental Health During the Covid-19 Pandemic: A Systematic Review and Meta-Analysis

**DOI:** 10.3389/fpubh.2021.793868

**Published:** 2022-02-01

**Authors:** Laura Marciano, Michelle Ostroumova, Peter Johannes Schulz, Anne-Linda Camerini

**Affiliations:** ^1^Faculty of Communication, Culture and Society, USI Università della Svizzera italiana, Lugano, Switzerland; ^2^Institute of Public Health, USI Università della Svizzera italiana, Lugano, Switzerland

**Keywords:** adolescence, social media, mental health, media addiction, well-being, review, Covid-19 pandemic

## Abstract

The Covid-19 physical distancing measures had a detrimental effect on adolescents' mental health. Adolescents worldwide alleviated the negative experiences of social distancing by spending more time on digital devices. Through a systematic literature search in eight academic databases (including Eric, Proquest Sociology, Communication & Mass Media Complete, Psychology and Behavioral Sciences Collection, PsycINFO, CINAHL, Pubmed, and Web of Science), the present systematic review and meta-analysis first summarized the existing evidence from 30 studies, published up to September 2021, on the link between mental health and digital media use in adolescents during Covid-19. Digital media use measures included social media, screen time, and digital media addiction. Mental health measures were grouped into conceptually similar dimensions, such as well-being, ill-being, social well-being, lifestyle habits, and Covid-19-related stress. Results showed that, although most studies reported a positive association between ill-being and social media use (*r* = 0.171, *p* = 0.011) and ill-being and media addiction (*r* = 0.434, *p* = 0.024), not all types of digital media use had adverse consequences on adolescents' mental health. In particular, one-to-one communication, self-disclosure in the context of mutual online friendship, as well as positive and funny online experiences mitigated feelings of loneliness and stress. Hence, these positive aspects of online activities should be promoted. At the same time, awareness of the detrimental effects of addictive digital media use should be raised: That would include making adolescents more aware of adverse mechanisms such as social comparison, fear of missing out, and exposure to negative contents, which were more likely to happen during social isolation and confinement due to the pandemic.

## Introduction

The Covid-19 pandemic and its related containment measures unavoidably affected mental health, which can be defined as “a state of well-being in which an individual realizes his or her own abilities, can cope with the normal stresses of life, can work productively and is able to make a contribution to his or her community” (1). Mental health is most affected during adolescence, when individuals enlarge their social sphere, establish a sense of autonomy, and make crucial decisions to achieve long-term goals (2). The concomitant maturation of social and cognitive control areas of the brain supports the progress of these skills, together with the exposure to and experience of appropriate contextual and social stimuli (3). Indeed, for adolescents, the social environment is important for developing essential brain functions, self-concept, and mental health in general (4). Hence, physical distancing measures introduced during the Covid-19 pandemic may have had a detrimental effect on youth development. Several studies already showed that adolescent age is a risk factor for diverse mental health problems, especially during epidemic outbreaks [e.g., (4–10)]. Particularly, social deprivation during a developmental period characterized by a high need for peer interaction likely augments negative consequences on mental health.

During the early months of the pandemic, many countries worldwide went into complete lockdown. Mental health of youth was threatened due to the shift toward distant learning, the closure of leisure environments, the decrease in outdoor activities, the impossibility to organize social events, and the increase of distress related to the pandemic. As measures were taken across the globe, their long-term effects on adolescents' mental health were unknown. To date, several reviews have summarized the immediate impact of the Covid-19 pandemic on the younger population. According to a review of ten studies (11), school closure contributed to anxiety, loneliness, stress, depressive symptoms, frustration in young people, together with higher indiscipline and hyperactive conduct. Similarly, also an increase in Body Mass Index and overweight was reported. A rapid narrative review of 15 articles (12) highlighted that pandemic and lockdown measures impacted young persons' mental health in particular, leading to a general decrease in psychological well-being followed by changes in sleep habits. Stressors were mainly linked to academic, economic, and social issues. Another review of six studies reported a general decrement in adolescents' quality of life during Covid-19, including the perception of physical, psychological, and social well-being (8). Overall, these reviews showed that young people were more vulnerable to psychological distress, highlighting the need for targeted interventions and psychological support.

Adolescents around the world alleviated the negative experiences of social distancing by spending more time online. A general increment in the use of digital technologies has been reported, especially of social media (13), with applications such as TikTok, Pinterest, Reddit, Facebook, Snapchat, Instagram, LinkedIn, and Twitter showing growth in active users ranging from 8 to 38% (14). Notably, teens reported staying connected with others via text messages (83%), phone calls (72%), social media and video chats (66%), instant messaging apps (48%), and, to a lesser extent, e-mails (37%) (15).

In line with this increment, a study on 5114 high school students from five countries (16) showed that more than 40% increased their social media time to stay connected with others since they could not meet in person. Similarly, Munasinghe et al. (17) reported augmented screen time – including social media, Internet, and smartphone use – together with diminished time for physical activity, decreased happiness, and more fast-food consumption. Focusing on the use of digital technology in 1,860 adolescents aged 12–18 years, Salzano et al. (18) reported that participants spent more than six hours a day on screens for educational purposes and from four-to-six hours a day for recreational activities. To note, adolescents reported that, on average, they sent and received over 100 messages per day. Not surprisingly, more frequent symptoms of smartphone dependency have been observed, especially among young females (19). The augmented time spent on digital technologies, particularly social media, might have alleviated feelings of loneliness and enhanced social connection. However, social media platforms also provided an overload of Covid-19 related information, where one-third of Covid-19 updates have been classified as fake (20), thus adding additional stress to the already worrisome situation. Accordingly, social media use has been identified as both a protective and risk factor for mental well-being during Covid-19 (21). This evidence should be interpreted in a larger, pre-pandemic context, where past reviews concluded that screen media use has negative but small effects on adolescents' health [e.g., (22, 23)] through various mechanisms such as upward social comparison and time displaced for other activities. This poses the question of whether the augmented use of screen media due to the pandemic may have exacerbated adverse outcomes by increasing social comparison and envy, displacing time for health-promoting activities such as sleep and exercising, and fostering cognitive distraction. Yet, screen media use could have also acted as a buffer, e.g., by initiating and maintaining social connections in times of limited face-to-face interactions or providing a way to get entertained. Additionally, adolescents may have used digital media as a coping tool to deal with the stress generated by the Covid-19 confinement by self-regulating their emotions using, for example, social media to escape ongoing worries and boost their mood (24).

To the best of our knowledge, no systematic synthesis on the link between digital media use, including social media and smartphone use, and adolescents' mental health during Covid-19 exists. Hence, the present systematic review and meta-analysis aims to fill this gap by focusing on the adolescent age, mental health, and digital media use during the Covid-19 pandemic.

## Methods

### Literature Search

On 16th April 2021, a systematic search was carried out in the titles and abstracts of scientific publications listed in eight academic databases, including Eric and Proquest Sociology (via Proquest), Communication & Mass Media Complete, Psychology and Behavioral Sciences Collection, PsycINFO, and CINAHL (via Ebscohost), Pubmed (via Medline and Proquest), Web of Science (via Clarivate Analytics). Key terms covered the population (e.g., “adolescent^*^”, “teen^*^”, “young^*^”), intervention/activity (e.g., “social media^*^, “screen time^*^”), outcome (e.g., “well-being^*^”, “psych^*^”, “mental^*^”), and context (e.g., “covid^*^”). They were combined using Boolean operators. The complete list of keywords and their combination is reported in Table 1.

**Table 1:** Complete list of keywords according to the PICO criteria.

Population:	adolescent* OR teen* OR tween* OR young* OR youth* OR child* student* OR adult*
	AND
Intervention/activity:	social network* OR social networking OR social media OR Facebook OR instagram OR snapchat OR SNS* OR screen time OR screen-time OR digital OR smartphone* OR Internet use*
	AND
Outcome:	well being* OR well-being* OR mood* OR anx* OR distress* OR stress* OR affect* OR life satisfaction* OR psychopatolog* OR psych* OR feeling* OR dependence* OR self-esteem* OR self-worth OR sleep* OR irritab* OR attention* OR inattent* OR fear* OR worry* OR insomnia* OR distract* OR panic* OR mental* OR health* OR behav* OR academic* OR school*
	AND
Context:	covid* OR covid-19* OR corona* OR pandemic* OR quarantine*

To exclude any duplicates, all entries were imported in Zotero, a reference management software. After duplicates were excluded, the remaining titles and abstracts were screened by two coders according to the predefined eligibility criteria. Cohen's kappa statistic (25) was calculated and used to measure inter-coder reliability. Discrepancies that emerged after full-text screening were resolved through a consensus meeting. Two additional hand searches were carried out on 15^th^ June 2021 and 15^th^ September 2021 to update the initial search due to the rapid rate of published works on the topic.

### Study Selection

According to the PICO [Population, Intervention, Comparison, and Outcome; (26)] definition of pre-specified eligibility criteria, we included articles with original data on a population aged 10 to 24 years (P) (27), including measures of (problematic) digital media as intervention (I), and mental health as the outcome (O), in the context of the Covid-19 pandemic. We did not include any comparison. Additionally, only studies published in peer-reviewed journals, written in English, and using a quantitative methodology with a cross-sectional or a longitudinal design were retained.

Articles were excluded if they were book chapters, pre-prints, conference papers, experimental studies, intervention studies, qualitative studies, studies focusing on gaming or cyberbullying, studies with no reference to Covid-19, as well as studies focusing on education, information-seeking behaviors, contact tracing, and clinical populations. Publications were also excluded if they reported only descriptive information of digital media use and mental health without linking the two concepts.

### Data Extraction

The following information was collected for each study: First author, year, and title of the paper, the country where the research was conducted, study design (cross-sectional or longitudinal), sample size, type of recruitment (online vs. other), type of sampling (random vs. convenience), % of male participants, age of participants, theoretical background, construct and measure of digital media use, construct and measure of mental health (including its different facets as *well*-being and *ill*-being), a brief description of the results, and raw data convertible into effect sizes.

### Quality Assessment of the Included Studies

The quality assessment of the included studies was carried out using the Strobe-checklist (28). In particular, for each study, we evaluated the quality of the information regarding the background/rationale, objectives, setting, participants, included variables, data sources/measurement, statistical methods, descriptive results, outcome data, key results, and limitations. The assessment of each study resulted in a total score from 0 to 11. A summary of the studies' quality can be found in Tables 2, 3. We also considered if the included studies used reliable measures for digital media use and well-being.

**Table 2:** Adapted STROBE checklist.

	**Dimension**	**Description**
Introduction	Background/ rationale	Explain the scientific background and rationale for the investigation being reported
	Objectives	State specific objectives, including all the hypotheses
Methods	Setting	Describe the setting, locations, and relevant dates, including periods of recruitment, exposure, follow-up, and data collection
	Participants	Give the eligibility criteria, and the sources and methods of selection of participants
	Variables	Clearly define all outcomes, exposures, predictors, potential confounders, and effect modifiers. Give diagnostic criteria, if applicable
	Data sources/ measurement	For each variable of interest, give sources of data and details of methods of assessment (measurement)
	Statistical methods	Describe all statistical methods. Explain how missing data were addressed
Results	Descriptive results	Give characteristics of study participants (e.g., demographic, clinical, social)
	Outcome data	Report numbers of outcome events or summary measures
Discussion	Key results	Summarise key results with reference to study objectives
	Limitations	Discuss limitations of the study, taking into account sources of potential bias or imprecision.
	Total	Total score

**Table 3:** Quality assessment of the included studies.

	**Introduction**		**Methods**					**Results**		**Discussion**		
**Study ID**	**Background/ rationale**	**Objectives**	**Settings**	**Participants**	**Variables**	**Data sources/ Measurement**	**Statistical methods**	**Descriptives data**	**Outcome data**	**Key results**	**Limitations**	**Total**
1	✓	✓	✓	✓	✓	✓	✓	✓	✓	✓	✓	11
2	✓	✓	✓	✓	✓	✓	✓	✓	✓	✓	✓	11
3	✓	✓	✓	✓	✓	✓	✓	✓	✓	✓	✓	11
4	✓	✓	✓	✓	✓	✓	✓	✓	✓	✓	✓	11
5	✓	✓	✓	✓	✓	✓	✓	✓	✓	✓	✓	11
6	✓	✓	✓	✓	✓	✓	✓	✓	✓	✓	✓	11
7	✓	✓	✓	✓	✓	✓	✓	✓	✓	✓	✓	11
8	✓	✓	✓	✓	✓	✓	✓	✓	✓	✓	✓	11
9	✓	✓	✓	✓	✓	✓	✓	✓	✓	✓	✓	11
10	✓	✓	✓	✓	✓	✓	✓	✓	✓	✓	X	10
11	✓	✓	✓	✓	✓	✓	✓	✓	✓	✓	✓	11
12	✓	✓	✓	✓	✓	✓	✓	✓	✓	✓	✓	11
13	✓	✓	✓	✓	✓	✓	✓	✓	✓	✓	✓	11
14	✓	✓	✓	✓	✓	✓	✓	✓	✓	✓	✓	11
15	✓	✓	✓	✓	✓	X	✓	✓	✓	✓	✓	10
16	✓	✓	✓	✓	✓	✓	✓	✓	✓	✓	✓	11
17	✓	✓	✓	✓	✓	✓	✓	✓	✓	✓	✓	11
18	✓	✓	✓	✓	✓	✓	✓	✓	✓	✓	✓	11
19	✓	✓	✓	✓	✓	X	✓	✓	✓	✓	✓	10
20	✓	✓	✓	✓	✓	✓	✓	✓	✓	✓	✓	11
21	✓	✓	✓	✓	✓	✓	✓	✓	✓	✓	✓	11
22	✓	✓	✓	✓	✓	X	✓	✓	✓	✓	✓	10
23	✓	✓	✓	✓	✓	✓	✓	✓	✓	✓	✓	11
24	✓	✓	✓	✓	✓	✓	✓	✓	✓	✓	✓	11
25	✓	✓	✓	✓	✓	✓	✓	✓	✓	✓	✓	11
26	✓	✓	✓	✓	✓	✓	✓	✓	✓	✓	✓	11
27	✓	✓	✓	✓	✓	✓	✓	✓	✓	✓	✓	11
28	✓	✓	✓	✓	✓	✓	✓	✓	✓	✓	✓	11
29	✓	✓	✓	✓	✓	✓	✓	✓	✓	✓	X	10
30	✓	✓	✓	✓	✓	✓	✓	✓	✓	✓	✓	11

✓* = present; X = absent*.

### Meta-Analytic Procedure

Due to the high heterogeneity of the included studies, meta-analytic syntheses were carried out only for studies including raw data convertible into effects sizes and similar investigated concepts. The “meta” (29) package in R statistical software was used for the meta-analysis. A Fisher's r-to-z transformation was calculated as a measure of effect size, and results were converted back to *r* correlation coefficients for interpretation. Conversion formulas (30, 31) were used when necessary to convert raw data to correlations. Several meta-analyses were carried out linking (i) social media use, (ii) screen-time, and (iii) media addiction to diverse mental health outcomes grouped into comparable categories. We interpreted pooled effect sizes of *r* = 0.10, *r* = 0.30, and *r* = 0.50 as small, medium, and large, respectively (32). An inverse-variance method with a random-effects model and Hartung-Knapp-Sidik-Jonkman adjustment (33) was used to adjust for study variability in sample sizes. Heterogeneity of results was calculated with the between-study-variance τ^2^, the restricted maximum-likelihood estimator (REML), and reported as I^2^ statistic (31, 34, 35). When possible (k = 10), additional meta-regression analyses were carried out to investigate the role of moderators, such as the age and gender of participants.

## Results

### General Overview

**Figure 1:**
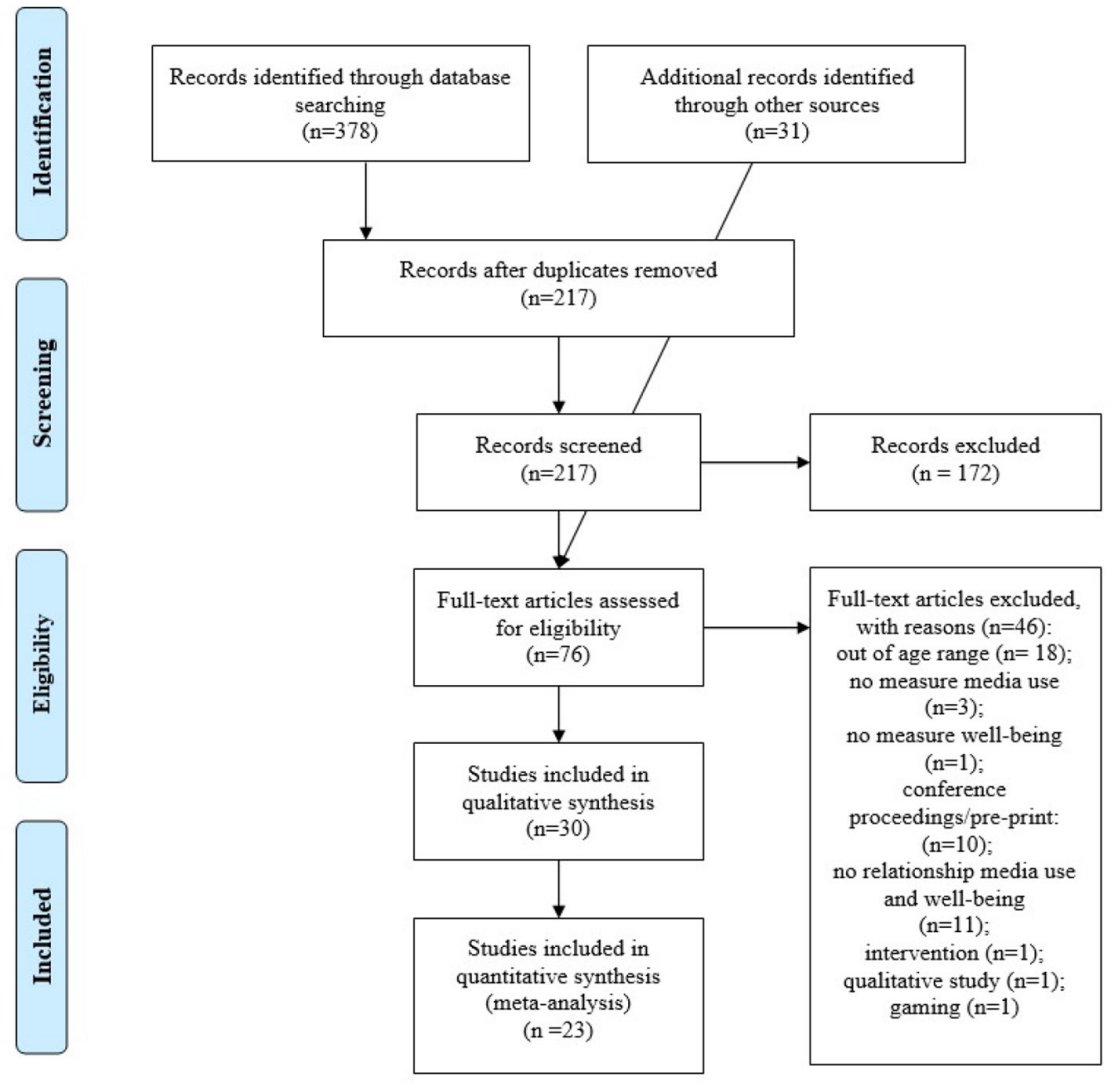
PRISMA flowchart.

From the initial database search, 378 records were obtained. After duplicates removal, the title and abstract of 217 records were screened independently by two coders based on the predefined eligibility criteria. Cohen's kappa as a measure of intercoder reliability was 0.80, indicating substantial agreement. After title and abstract screening, 45 articles were retained. Another 31 articles were identified through the two additional hand searches, adding up to 76 articles for full-text screening. Of these, 30 articles were retained in the systematic review and a subset of 23 in the meta-analysis (see PRISMA flowchart in Figure 1).

The included studies were mainly conducted in Asia (*n* = 12) and Europe (*n* = 11). Few were conducted in Oceania (*n* = 3), America (*n* = 2), and Middle East (*n* = 1). One study (36) collected data from Italy, Argentina, and United Kingdom. Six studies adopted a longitudinal design, ranging from 14 days (37) to twelve months (38). In all the studies, data were collected through online questionnaires, and three studies made use of a random sampling procedure. The median sample size was 760, ranging from 102 to 13,525, with one longitudinal study including 1,64,101 participants at the first time point of data collection (39). In general, females were slightly over-represented (mean = 60%). Participants' mean age was 17.75 years (ranging from 9.50 to 25.5).

**Figure 2:**
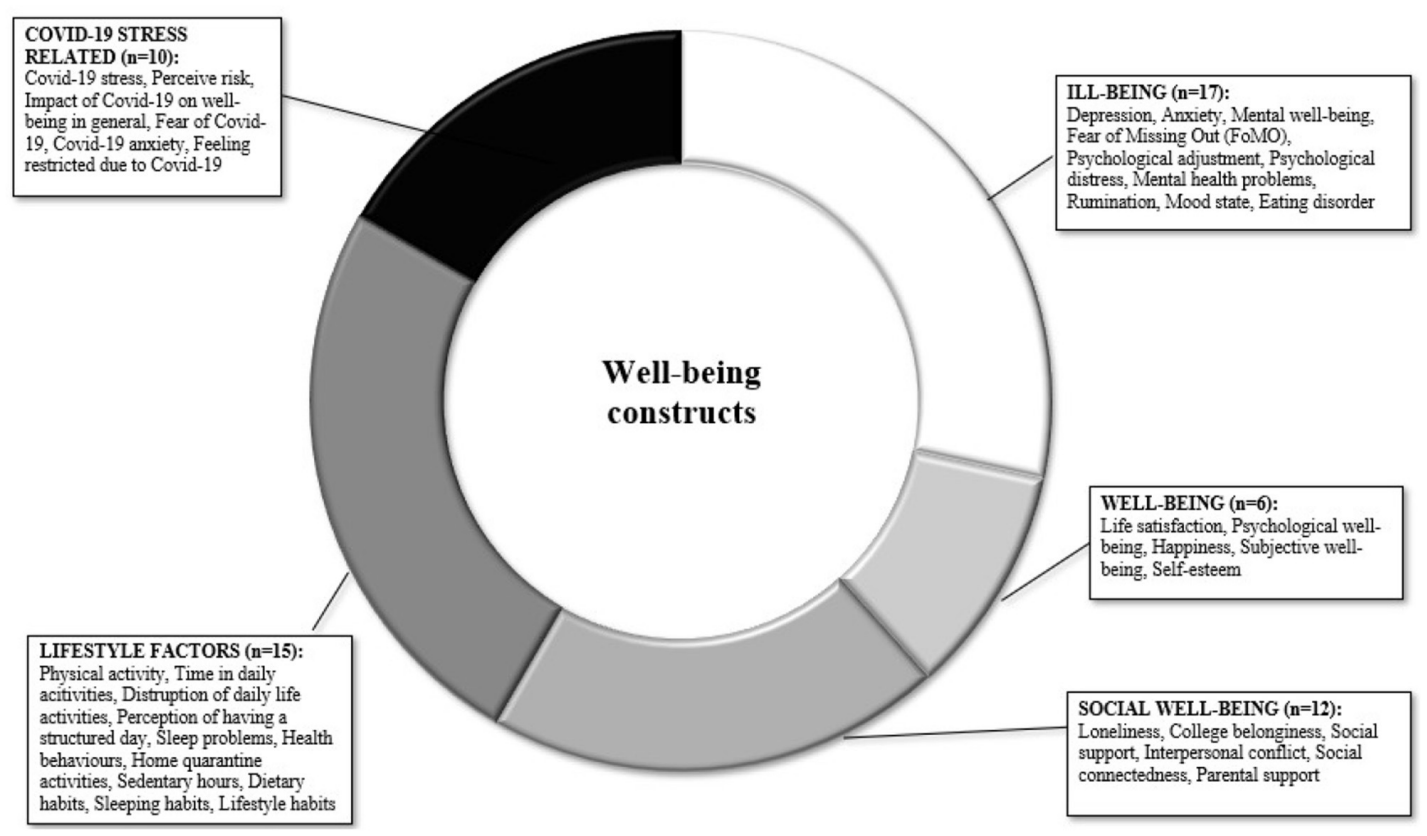
Graphic representation of well-being investigated constructs.

**Figure 3:**
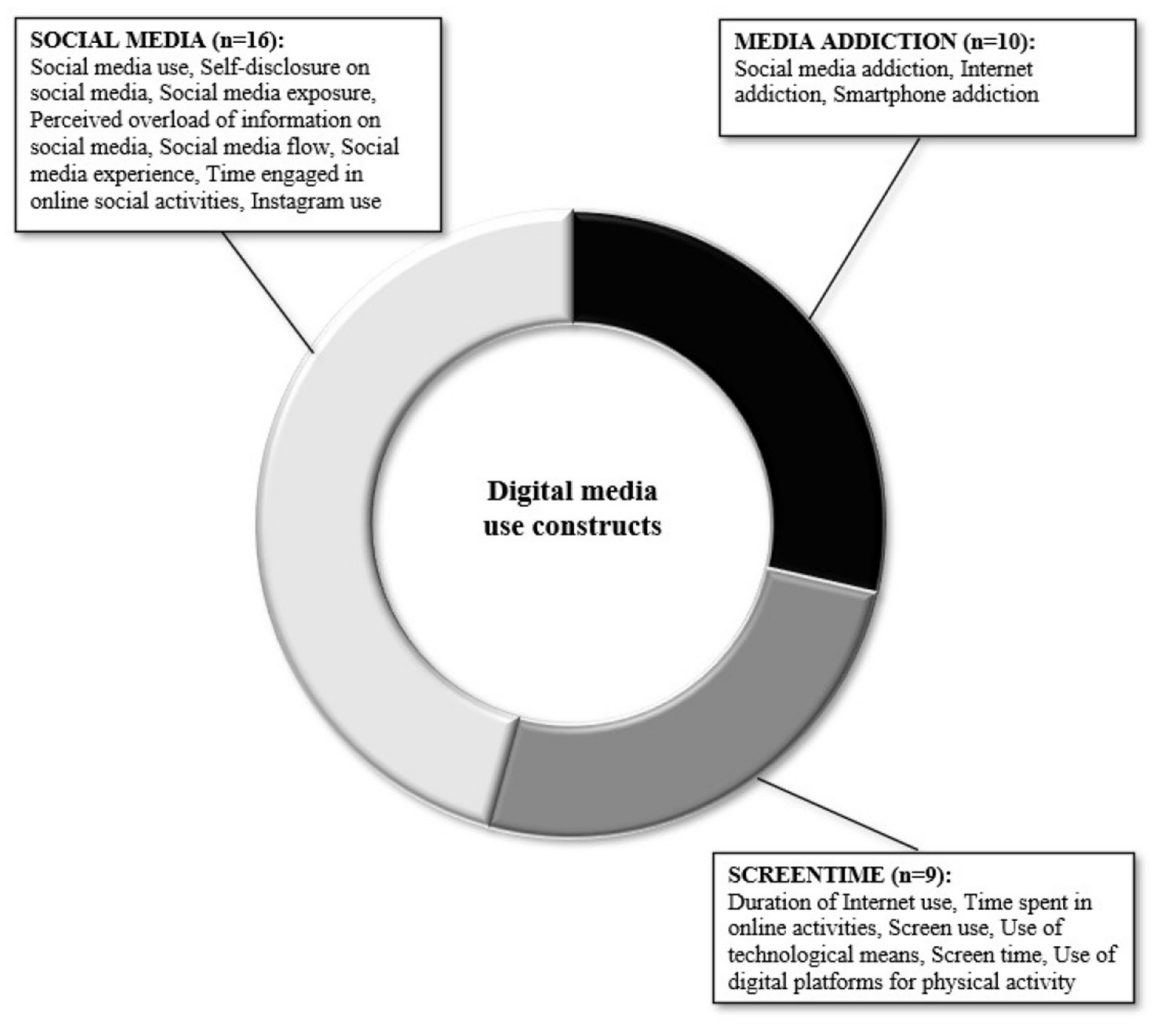
Graphic representation of digital media use investigated constructs.

Included studies mainly assessed social media use (*n* = 16), screen time (*n* = 10; including time spent on different devices, change in screen time, and type of usage), and media addiction (*n* = 9; including measures of Internet and social media addiction). Mental health was measured in terms of *ill*-being (*n* = 17, i.e., psychopathological problems such as symptoms of depression, anxiety, mood disorder, ruminative thoughts), *well*-being (*n* = 6, including life satisfaction, optimism, happiness), social well-being (*n* = 12, covering the quality of social relationships, social support, interpersonal conflict, and loneliness), lifestyle habits (*n* = 15, including physical activity, sleep, smoking, nutrition, and everyday health routines), and Covid-19-related stress (*n* = 10, covering distress, fear, and worries due to the Covid-19 pandemic). For a summary of the investigated concepts, see Figures 2, 3.

A summary of the study characteristics can be found in Tables 4–6. Applying the Strobe checklist, all studies were of very good quality, except for three studies lacking detailed information on data sources and measurement (55, 58, 66) and two studies with insufficient recognition of their study limitations (48, 64).

**Table 4:** Summary of the included studies.

**ID**	**Author and Year**	**Title**	**Country**	**Study design**	**Duration**	**Data collection**	**N**	**Sampling**	**Males**	**Age**	**Theory**
1	Arend et al. (37)	Increased screen use on days with increased perceived COVID-19-related confinements - a day level ecological momentary assessment study	Germany, Austria	L	14 days	online	102	R	18.6%	25.5	–
2	Ellis et al. (40)	Physically isolated but socially connected: psychological adjustment and stress among adolescents during the intial COVID-19 crisis	Canada	C		online	1,054	C	21.9%	16.68	–
3	Li et al. (41)	The impact of COVID-19 on the loves and mental health of Australian adolescents	Australia	C		online	760	C	19%	14.8	–
4	Liu et al. (42)	COVID-19 information overload and generation Z's social media discontinuance intention during the pandemic lockdown	United Kingdom	C		online	322	C	38.80%	18-25	Stimulus-organism-response (S-O-R) model
5	Zhao et al. (43)	COVID-19 stress and addictive social media use (SMU): mediating role of active use and social median flow	China	C		online	512	C	37.5%	22.12	Addictive social media use, Theory of basic psychological needs
6	Arslan et al. (44)	Coronavirus Anxiety and Psychological Asjustment in College Students: Exploring the Role of College Belongingness and Social media Addiction	Turkey	C		online	315	C	23%	21.65	–
7	Zhen et al. (45)	College students coping with COVID-19: stress-buffering effects of self-discolsure on social media and parental support	United States	C		online	215	C	21%	20.5	Stressful life events, Social penetration theory
8	Dong et al. (46)	Internet Addiction and Related Psychological Factors Among Children and Adolescents in China During the Coronavirus Disease 2019 (COVID-19) Epidemic	China	C		online	2,050	C	52%	12.34	–
9	Vall-Roqué et al. (47)	The impact of COVID-19 lockdown on social network sites use, body image disturbance and self-esteem among adolescent and young women	Spain	C		online	1,620	C	0%	14-24	–
10	Sheoran et al. (48)	Prevalence of psychological distress among adolescents in relation to internet addiction during COVID-19 times	India	C		online	300	C	50%	15.57	–
11	Hong et al. (49)	Social media exposure and college students' mental health during the outbreak of covid-19: the emdiating role of rumination and the moderating role of mindfulness	China	C		online	439	C	58.10%	20.37	Health belief model, Integrated model of ruminative response style, Diathesis-stress model
12	Magson et al. (38)	Risk and protective factors for prospective changes in adoelscent mental health during the COVID-19 pandemic	Australia	L	12 months	online	248	C	50%	14.4	–
13	Li et al. (50)	Mental health among college students during the COVID-19 pandemic in China: a 2-wave longitudinal survey	China	L	2.5 months	online	T1: 164 101; T2: 68 658	C	37.40%	college year (freshman, sophomore, junior, senior, and graduate)	–
14	Chambonn -iere et al. (51)	Effect of the covid-19 lockdown on physical activity and sedentary behvaiors in french children and adolescents: new results from the ONAPS national survey	France	C		online	6,491	C	38.80%	6-17	–
15	Islam et al. (52)	Problematic internet use among yound and adult population in Bangladesh: Correlates with lifestyle and online activities during the COVID-19 pandemic	Bangladesh	C		online	13,525	C	61.30%	23.7	–
16	Parker et al. (53)	The use of digital platforms for adult's and adolescents' physical activity during the COVID-19 pandemic (our life at home): survey study	Australia	C		online	963	R	28.90%	16.2	–
17	Dragun et al. (54)	Have lifestyle habits and psychilogical well-being changed among adolescents and medical students due to COVID-19 lockdown in Croatia?	Croatia	C		offline/online	T1: 1326; T2: 531	C	40%	18	–
18	Fumagalli et al. (36)	Centennials, FOMO, and loneliness: an investigation of the impact of social networking and messaging/VoIP apps usage during the initial stage of the coronavirus pandemic	Italy, Argentina, United Kingdom	L	1 month	online	334	C	30.20%	21.5	Evolutionay theory of loneliness
19	Magis-Weinberg et al. (55)	Positive and Negative Online Experiences and Loneliness in Peruvian Adolescents During the COVID-19 Lockdown	Latin America	L	3 months	online	735	C	38.80%	13.25	–
20	Rens et al. (56)	Mental distress and its contributing factors among young people during the first wave of COVID-19: a belgian survey study	Belgium	C		online	2,008	R	21.91%	22.27	–
21	Xiao et al. (57)	Physical activity, screen time and mood disturbance among chinese adolescents during COVID-19	China	C		online	1,680	C	51.30%	7-12	–
22	Nomura et al. (58)	Cross-sectional survey of depressive symptoms and suicide-related ideation at a japanese national unviersity during the COVID-19 stay-home order	Japan	C		online	2,449	C	58%	20	–
23	Hudimova et al. (59)	The impact of social media on young web user's psychological well-being during the COVID-19 pandemic progression	Ukraine	C		online	254	C	NA	16-21	–
24	Cauberghe et al. (60)	How adolescents use social media to cope with feelings of loneliness and anxiety during COVID-19 lockdown	Belgio	C		online	2,165	C	34.4%	15.51	Mood management theory
25	Pigaiani et al. (61)	Adolescent lifestyle behaviors, coping strategies and subjective wellbeing during the COVID-19 pandemic: an online student survey	Italy	C		online	306	C	72.90%	18.1	–
26	Islam et al. (52)	Problematic Smartphone and Social Media Use Among Bangladeshi College and University Students Amid COVID-19: The Role of Psychological Well-Being and Pandemic Related Factors	Bangladesh	C		Online	5,511	C	58.90%	21.2 (1.7)	–
27	Chen et al. (62)	Internet-Related Behaviors and Psychological Distress Among Schoolchildren During the COVID-19 School Hiatus	China	C		Online	2,026	C	50.10%	10.71 (1.07)	Interaction of Person- Affect-Cognition-Execution (I-PACE) model
28	Fung et al. (63)	Problematic Use of Internet-Related Activities and Perceived Weight Stigma in Schoolchildren: A Longitudinal Study Across Different Epidemic Periods of COVID-19 in China	China	L	6 months	Online	T1: 550; T2: 543; T3: 489	C	51%	11.60 (.74)	Components model of addiction
29	Hayran et al. (64)	Well-Being and Fear of Missing Out (FOMO) on Digital Content in the Time of COVID-19: A Correlational Analysis among University Students	Europe	C		online	178	C	62	21.35 (1.82)	–
30	Siste et al. (65)	Implications of COVID-19 and Lockdown on Internet Addiction Among Adolescents: Data From a Developing Country	Indonesia	C		online	2,932	C	21,3%	17.38 (2.24)	–

*Study design (C, correlational; L, longitudinal): Duration If longitudinal duration in months (between first and last wave); N, analytical sample size; Sampling (Type of sampling procedure) (R, random; C, convenient; other, specify); Age (Mean, Standard deviation or Range, if Longitudinal, M and SD at T1 are reported)*.

**Table 5:** Digital media and well-being constructs investigated.

**ID**	**Author and Year**	**Media**	**Media construct**	**Measures**	**Reliability**	**Media in the model**	**Mental health**	**Mental health construct**	**Measures**	**Reliability**	**Mental health in the model**
1	Arend et al. (37)	SCREEN	Screen time	Watching TV, Social media use, News consumption, Video games, Internet use	5 items	O	COVID/ LIFESTYLE	1) Feeling restricted due to the pandemic; 2) Perception of having a structured day	1) Socially and in the work environment; 2) Very/lowly structured day	Nr	P, MOD
2	Ellis et al. (40)	SOCIAL	Social media use	How many hours per day you spent on social media 6 months before and during the pandemic	2 items	P	COVID, LIFESTYLE, ILLBEING, SOCIAL	1) COVID-19 stress; 2) Time in daily activities; 3) Depression; 4) Loneliness; 5) Physical activity	1) Fear of the spread of the Covid, Swine Flu Anxiety scale; 2) How have your days gone in the last 3 weeks since the pandemic, how much time spent in the single activities; 3) Brief Symptom Inventory; 4) UCLA Loneliness Scale; 5) Godin Leisure-time Exercise Questionnaire	1) 8 items, Swine Flu Anxiety Scale: Cronbach's alpha = 0.60; 2) Nr; 3) 6 items, Cronbach's alpha = 0.88; 4) 8 items, Cronbach's alpha = 0.81; 5) Si	O
3	Li et al. (41)	SCREEN	Use of technological means	Screen time (not including time for online teaching), How much time used for exchanges with friends and family, How technology use has changed since the pandemic	3 items	P	COVID, LIFESTYLE, WELLBEING, ILLBEING	1) Covid exposure, perceived risk, and changes in behavior; 2) Impact of Covid on physical and mental well-being, school/education, and relationships; 3) Lifestyle; 4) Mental well-being and well being	1) Covid exposure, perceived risk, changes in behavior (social distance, hygiene,.); 2) Physical and mental well-being, school and education, relationships with others, family functioning; 3) Exercise, insomnia severity index, UCLA Loneliness Scale, uncertainty about the future; 4) Kessler-6, seven-item Warwick Edinburgh Mental Well-being Scale, Body Preoccupation Scale of the Illness Attitude Scales	1) 9 items; 2) 2 items for family functioning, 3) 7 items for sleep, Si for loneliness, Si for uncertainty about the future; 4) Nr, K6, 7 items SWEMWS, 3 items BPSIAS	O
4	Liu et al. (42)	SOCIAL	1) Perceived COVID-19 information overload on social media as environmental stimulus; 2) Social media fatigue; 3) Social media discontinuance intention	Ad hoc items	1) 3 items, Cronbach's alpha =0.834; 2) 3 items, Cronbach's alpha =0.871; 3) 4 items, Cronbach's alpha =0.813	P, MED	COVID, SOCIAL	1) Fear of missing out; 2) Fear of Covid-19	Items used by another study	1) 3 items, Cronbach's alpha =0.884; 2) 4 items, Cronbach's alpha =0.904	O, MOD, MED
5	Zhao et al. (43)	ADDICTION, SOCIAL	1) Addiction to social media use; 2) Active social media use; 3) Social media flow; 4) Hours of social media use	1) Brief version of Bergen Facebook Addiction Scale; 2) 4 items adapted from the assessment tool developed by Brailovskaia and Margraf; 3) Modified version of “Facebook flow” developed by Brailovskaia et al.; 4) Method of Lin et al.	1) 6 items, Cronbach's alpha = 0.84; 2) 4 items, Cronbach's alpha = 0.78; 3) 11 items, Cronbach's alpha = 0.82, 4) 1 item	O, MED	COVID	Covid-19 stress	SARS-related stress by Main et al.	10 items	P
6	Arslan et al. (44)	ADDICTION	Social media addiction	Bergen Social Media Addiction Scale	6 items	MED	COVID, SOCIAL, ILLBEING	1) Coronavirus anxiety; 2) College belongingness; 3) Psychological Adjustment	1) Coronavirus Anxiety Scale; 2) College Belongningness Questionnaire; 3) Brief Adjustment Scale-6	1) 5 item; 2) 10 item; 3) 6 item	O, MED, P
7	Zhen et al. (45)	SOCIAL	1) Peripheral disclosure on social media; 2) Core disclosure on social media	1) Degree to which participant willing to share general information on social media; 2) Degree to which participant willing to share private information and with whom on social media	1) 5 items, Cronbach's alpha = 0.71; 2) 2 items, Cronbach's alpha = 0.82	P	COVID, SOCIAL, LIFESTYLE	1) Disruption of daily life due to the pandemic; 2) Self-revelation; 3) Support from parents; 4) Perceived stress due to Covid	1) 7-point Likert scale; 2) 7 items from Osatuyi et al. 2018; 3) shortened version of MOS Social Support Survey; 4) Perceived Stress Scale	1) 5 items, Cronbach's alpha = 0.71; 2) 34 items; 3) 10 items, Cronbach's alpha = 0.94; 4) 10 items, Cronbach's alpha = 0.88	O
8	Dong et al. (46)	SCREEN, ADDICTION	1) Screen time; 2) Internet use; 3) Internet addiction	1) Primary electronic devices used; 2) recreational use, nightime use; 3) Chinese version of Young's Internet Addiction Test (IAT)	1) Multi item; 2) 20 items, Cronbach's alpha > 0.82	P	ILLBEING	1) Depression, Anxiety, Stress	Chinese version of Depression, Anxiety, and Stress Scale (DASS-21)	21 items	O
9	Vall-Roqué et al. (47)	SOCIAL	Social media use	Frequency of instagram, youtube, tiktok, twitter, and facebook usage before and during the lockdown, What profiles the participant follows on instagram before and during the lockdown	4 items	P	ILLBEING, WELLBEING	1) Eating disorder and drive for thinness; 2) Self-esteem	1) The drive for thinnes and body dissatisfaction subscales of the Eating Disorders Inventory, latter subscale; 2) Rosenberg Self-esteem Scale	1) 7 items, 10 items, drive for thinness Cronbach's alpha = 0.92 e body dissatifscation Cronbach's alpha = 0.90; 2) 10 item, Cronbach's alpha = 0.85	O
10	Sheoran et al. (48)	ADDICTION	Internet addiction	Internet addiction test	20 items, Cronbach's alpha between 0.54 and 0.82	O, P	ILLBEING	Psychological distress	Psychological distress subscale of mental health inventory	38 items, Cronbach's alpha = 0.94	O, P
11	Hong et al. (49)	SOCIAL	Social media exposure	How much participants are exposed to information regarding covid on the 6 most used social media in China	6 items, Cronbach's alpha =0.67	P	WELLBEING, ILLBEING	1) Mindfulness; 2) Rumination; 3) Psychological distress	1) Chinese version of the Child and Adolescent Mindfulness Measure; 2) event- related rumination inventory; 3) Kessler Psychological Distress Scale	1) 10 items, Cronbach's alpha # =0.90; 2) 10 items, Cronbach's alpha =0.93; 3) 10 items, Cronbach's alpha =0.94	O, ME
12	Magson et al. (38)	SOCIAL	Social media exposure	Social media exposure	2 items	P, MO	ILLBEING, COVID, SOCIAL	1) General anxiety; 2) Depressive symptoms; 3) Life satisfaction; 4) Covid-19 related distress; 5) Disruption to schooling; 6) Interpersonal conflict; 7) Media exposure; 8) Social connectedness; 9) Adherence to Covid-19 Australian government stay-at-home directive	1) Generalized anxiety subscale of the Spence Children's Anxiety Scale; 2) Short Mood and Feelings Questionnaire-child version; 3) Student's Life Satisfaction Scale; 4) How much perceived stress due to Covid; 5) Perception of homeschooling and distance learning; 6) Conflicts with parents and siblings; 7) Social Connectedness Scale; 8) How often left home	1) Cronbach's alpha = T1- 0.86, T2- 0.87; 2) 13 items, Cronbach's alpha = T1- 0.91,T2- 0.93, 3) 9 items; Cronbach's alpha = T1- 0.91, T2- 0.92, 4) 18 items; Cronbach's alpha = T2- 0.91; 5) Nr; 6) Cronbach's alpha = 0.91; 7) 1 item	O
13	Li et al. (50)	SOCIAL	Social media exposure	Hours exposed to info regarding covid the previous week on social media	3 items	P	ILLBEING, COVID, SOCIAL, LIFESTYLE	1) Health behavior; 2) Mental health problems; 3) Exposure to Covid-19 epidemic and related factors; 4) Psychosocial factors	1) Lifetime cigarette smoking, alcohol use and daily physical activity; 2) Impact of Event Scale-6, Patient Health Questionnaire, Chinese version of the Generalized Anxiety Disorder Scale; 3) Whether participants became sick with Covid or were suspected sick in their vicinity, severity of the pandemic in their region, exposure to social media; 4) Scale of Perceived Social Support, role of family	1) Nr, 2) Acute stress symptoms: 6 items, Cronbach's alpha = 0.80 and 0.82, Depressive symptoms: Cronbach's alpha =0.88 and 0.91, Anxiety symptoms: 7 items, Cronbach's alpha = 0.92 and 0.93	O
14	Chambonniere et al. (51)	SCREEN	Screen time	Media hours spent before and during the lockdown	Nr	O,P	LIFESTYLE	1) Physical activity; 2) Sedentary hours	1) Hours of physical activity before and during lockdown; 2) Sedentary activities before and during the lockdown, what they did before the lockdown	Nr	O,P
15	Islam et al. (52)	ADDICTION, SCREEN	1) Duration of internet use; 2) Problematic internet use	1) Average hours spent on the internet; 2) What participants did on the internet, IDS9-SF	9 items, Cronbach's alpha: 0.85	O	LIFESTYLE	Lifestyle-related behaviors	Smoking status, Sleeping hours, Physical exercise, Doing household chores	4 items	P
16	Parker et al. (53)	SCREEN	Use of digital platforms for physical activity	Frequency and duration of use of digital physical activity platforms, What type of online platform	Nr	P	LIFESTYLE	Compliance with recommendations regarding physical activity	How many days per week in the past month, for 30/60 min, whether at home or in the gym before the pandemic	Nr	O
17	Dragun et al. (54)	SCREEN	Screen time	Computer/tablet/cell phone/TV hours usage per day	3 items	P	LIFESTYLE, WELLBEING, ILLBEING	1) Dietary habits; 2) Sleeping habits; 3) Sedentary activity; 4) Psychological well-being; 5) Lifestyle habits	1) Mediterranean diet adherence, consumption of sweet drinks and processed meat/fish, frequency of breakfast consumption, snacking habits while studying/TV; 2) Wake up time and when you go to bed, how you feel after waking up; 3) Sedentary activities and frequency of physical activity; 4) Perceived Stress Scale, happiness, anxiety and optimism about the future; 5) Consumption of fruits and vegetables, body weight,...	1) 3 items; 2) 4 items; 3) Si; 4) 6 items; 5) 7 items	O
18	Fumagalli et al. (36)	SOCIAL	1) Screen time; 2) Social media use	Trace data of total time spent on the smartphone, time using apps of various categories, time spent on a single installed application, set a limit for a certain app or not	Nr	P	SOCIAL	1) FOMO; 2) Loneliness; 3) Personality characteristics	1) 3-item measure; 2) UCLA Loneliness Scale; 3) 7-point scale	1) 3 items, Cronbach's alpha =0.39; 2) 8 items, Cronbach's alpha = 0.82; 3) 10 items	O
19	Magis-Weinberg et al. (55)	SOCIAL, SCREEN	1) Social media experience; 2) Screen time	1) Online social experience measure; 2) Hours spent from 0 to 8 with video games, watching TV, pc or cell phone	1) 11 items, Cronbach's alpha pos = 0.85, Cronbach's alpha =0.79; 2) 1 item	P	SOCIAL	1) Loneliness; 2) Perceived family support	1) UCLA Loneliness Scale; 2) Multidimensional Scale of Perceived Social Support	1) Cronbach's alpha =0.79; 2) 12 items, Cronbach's alpha = 0.88,0.90,0.86,0.90	O
20	Rens et al. (56)	SOCIAL	Social media use	Daily social media usage in hours	2 items	P	SOCIAL, COVID, LIFESTYLE	1) Mental distress; 2) Social support; 3) Exposure to Covid-19; 4) Home activities; 5) Change in everyday life; 6) Experiencing loneliness	1) General Health Questionnaire; 2) Oslo Social Support Scale, 3) Have/had Covid, has/had a relative with Covid; 4) Change in time spent at home before and during the pandemic; 5) Visiting friends and relatives, going out to drink and eat, physical activity, hobbies and activities at home; 6) UCLA 3-item Loneliness Scale	1) 12 items; 2) 3 items; 3) Si; 4) Nr; 5) 5 items; 6) 3 items	O
21	Xiao et al. (57)	SCREEN	Screen time	Hours spent online for school and other reasons	2 items	P	SOCIAL, ILLBEING, LIFESTYLE	1) Mood state; 2) Physical activity; 3) Conflicts with parents	1) Chinese version of the Mood Profile, Total Mood Disorder; 2) Questions about leisure exercise; 3) Number of conflicts with parents	1) Nr; 2) Nr; 3) Si	O
22	Nomura et al. (58)	SOCIAL	1) Social media use; 2) Social media disorder	Communication through text, audio and video on various social networks	Si	P	SOCIAL, LIFESTYLE, ILLBEING	1) Social support; 2) Exercise; 3) Depressive symptoms	1) Presence of someone to talk to about your concerns; 2) Measurement of the intensity of physical activity performed, how long in minutes,...; 3) Japanese version of PHQ-9	1) 2 items; 2) Si; 3) 9 items	O
23	Hudimova et al. (59)	SOCIAL, ADDICTION	Social media use	Social media disorder scale, Experience using social media, Time spent on social media per day, Social media disorder scale, Number of profiles	Cronbach's alpha = 0.82	P	WELLBEING, LIFESTYLE, ILL-BEING	Psychological well-being	Sleep changes, worry, tolerance, feeling connected during quarantine, avoidance behaviors, conflict, loneliness, Warwick-Edinburgh Mental Wellbeing Scale	Nr	O
24	Cauberghe et al. (60)	SOCIAL	Social media use	Brief-coping scales, How social media used to manage crisis, Coping strategies to manage lack of social relationships and manage mood	14 items, Cronbach's alpha = 0.722, 0.765	MED	WELL-BEING, ILLBEING, SOCIAL	1) Happiness; 2) Anxiety; 3) Loneliness; 4) Happiness/depression	1) Three items of the Center for Epidemiological Studies Depression Scale; 2) General Anxiety Disorder Scale, 3) 6-item scale (RULS-6); 4) Feelings of sadness/happiness	1) 3 items; 2) 7 items, Cronbach's alpha = 0.868; 3) 6 items, Cronbach's alpha = 0.768; 4) 3 items, Cronbach's alpha =0.811	O, P
25	Pigaiani et al. (61)	SOCIAL	1) Video games; 2) Social media use	1) Playing video games; 2) Using social networks to stay in touch with family, acquiring new skills through social networks	Si	P	WELLBEING, LIFESTYLE	1) Subjective well-being; 2) Lifestyle and coping skills	1) Difficulty staying at home, impact of the pandemic experience on the person, changes in psychological well being, anxiety about the situation, sleep abstinence; 2) organization of the day, activities performed and how they have changed, relationship with family, distance learning	Nr	O
26	Islam et al. (52)	ADDICTION	1) Problematic social media use; 2) Problematic smartphone use	1) Bergen Social Media Addiction Scale (BSMAS); 2) Smartphone Application Based Addiction Scale (SABAS)	1) 6 items, Cronbach's alpha = 0.80; 2) 6 items, Cronbach's alpha =0.85	O	ILLBEING, LIFESTYLE	1) Depressive symptoms; 2) Anxiety symptoms; 3) Lifestyle Measures; 4) Home Quarantine Activities Measures during Covid-19	1) Patient Health Questionnaire (PHQ-9); 2) Generalized Anxiety Disorder (GAD-7); 3) Items measuring sleep, physical exercising, smoking cigarettes, and alcohol consumption; 4) Items on the engagement in frequent activities during the pandemic, including home quarantine regular/frequent activities (i.e., academic/other studies, social-media use, watching television, household chores, and professional activities).	1) 9 item, Cronbach's alpha =0.89; 2) 7 item, Cronbach's alpha =0.91; 3) Nr; 4) Nr	O
27	Chen et al. (62)	ADDICTION, SCREEN	1) Social media addiction; 2) Smartphone addiction; 3) Gaming addiction; 4) Time spent in online activities	1) Bergen Social Media Addiction Scale (BSMAS); 2) Smartphone Application-Based Addiction Scale (SABAS); 3) Internet Gaming Disorder Scale-Short Form (IGDS-SF9); 4) Ad hoc questions	1) 6 items, Cronbach's alpha =0.875; 2) 6 items, Cronbach's alpha =0.882; 3) 9 items, Cronbach's alpha =0.922; 4) si	O, MED	ILLBEING	Psychological distress (symptoms of anxiety, depression, and stress)	Depression, Anxiety, Stress Scale-21 (DASS-21)	21 item, Cronbach's alpha =0.820, 0.778, 0.813	P
28	Fung et al. (63)	ADDICTION	1) Social media addiction; 2) Smartphone addiction	1) Bergen Social Media Addiction Scale (BSMAS); 2) Smartphone Application-Based Addiction Scale (SABAS)	1) 6 items, Cronbach's alpha =0.83, 0.88, 0.89; 2) 6 items, Cronbach's alpha =0.78, 0.90, 0.88	P	ILLBEING	Psychological distress (symptoms of anxiety, depression, and stress)	Depression, Anxiety, Stress Scale-21 (DASS-21)	21 items, Cronbach's alpha =0.93, 0.91, 0.93	O
29	Hayran et al. (64)	SOCIAL	1) Time they engaged in online social activities (such as virtual gatherings with family and friends, watching real-time Instagram concerts or interviews) since the beginning of the pandemic; 2) Whether the time they spent on social media increased during the pandemic	Ad-hoc scales	10 items, 2 items, 2 items, 4 items	P	ILLBEING	FOMO	State and trait FOMO	10 items, Cronbach's alpha =0.82	O
30	Siste et al. (65)	ADDICTION	1) Internet addiction	Internet Addiction Diagnostic Questionnaire	44 items, Cronbach's alpha =0.979	O	ILLBEING	1) Psychopatological problems; 2) Sleep problems	1) Strengths and Difficulties Questionnaire; 2) Pittsburgh Sleep Quality Index	1) 25 items, Cronbach's alpha =0.773; 2) 24 items, Cronbach's alpha =0.79	P

*Media (SCREEN, screen time in general; ADDICTION, Internet, social media, smartphone addiction; SOCIAL, social media use); Reliability (number of items/inter-item correlation/Cronbach's alpha; nr, not reported; si, single item scale); Media and Mental health in the model (P, Predictor, Med, Mediator, Mod, Moderator, O, Outcome); Mental health (WELLBEING, in a positive sense such as self-esteem, life satisfaction; ILLBEING, anxiety, depression, other symptoms; COVID, stress related to Covid-19; SOCIAL, social well-being; LIFESTYLE, physical activity, food, sleep, etc.)*.

**Table 6:** Description of results.

**ID**	**Author and Year**	**Brief description of results**
1	Arend et al. (37)	Results showed that participants reported increased screen use during leisure time, mostly social media and television watching, followed by news consumption, other internet usage, and gaming. Experienced work confinements were positively associated with social media usage. Further, work confinements were positively associated with gaming in males and with news consumption, especially in individuals living alone. Social confinements were positively associated with watching television especially in younger participants and with social media consumption in younger participants. Higher experienced day structure was related to less television watching, gaming, and internet surfing but more news consumption.
2	Ellis et al. (40)	Frequency of social media use increased during the pandemic and physical activity decreased. Social media use increased, with 48% of participants using social media for more than 5 hours. Although social media use is nearly universal among youth (95% of teens reported at least 30 min per day), results showed a substantial increase in the time spent using a variety of social media platforms (e.g., Instagram, Snapchat, TikTok,) during the initial call to stay at home. Shockingly, over 12% of adolescents reported using social media more than 10 hrs a day. Analyses showed virtual time with friends related to higher depression but lower loneliness, beyond reported COVID-19 stress.
3	Li et al. (41)	Most youth reported: worsening family stress, decreased/maintained physical activity, higher technology use. 93% say they have some level of worry about the future and 40.1% show clinical symptoms of illness anxiety. Participants with previously diagnosed anxiety or depression showed lower levels of physical activity, higher technology use, worse sleep quality, higher levels of loneliness and uncertainty, lower levels of psychological stress, lower levels of mental well-being, and anxiety about health. Use of technology to feel with other people is associated with better overall well-being and lower levels of loneliness.
4	Liu et al. (42)	The results indicate that the perceived COVID-19 information overload on social media increased social media fatigue and fear of COVID-19.The enormous amounts of complex information related to COVID-19 exceeded the information-processing capacity of the members of Gen Z and further hindered their ability to develop an unbiased assessment of COVID-19, which led to a higher level of fear of the coronavirus pandemic. Results suggest that fear of missing out acts as a moderator that weakens the associations between the inner psychological states of Gen Z social media users (i.e., social media fatigue and fear of COVID-19) and their social media discontinuance intention. Users who are high in FoMO tend to derive more benefits from social media use, particularly during the time of lockdown.
5	Zhao et al. (43)	The results showed that COVID-19 stress was positively associated with tendencies toward addictive SMU. Path analyses revealed that this relationship was significantly serially mediated by active use and social media flow, with SMU time being controlled. Our findings suggest that individuals who experience more COVID-19 stress are at increased risk of addictive SMU that may be fostered by active use and flow experience. Therefore, the significant indirect path from COVID-19 stress to addictive SMU via active use implies that excessive active use acts as a maladaptive coping strategy in the time of the COVID-19 crisis.
6	Arslan et al. (44)	Higher experience of coronavirus anxiety was associated with lower college belongingness and higher psychological adjustment problems. Adjustment via college belongingness only occurred when social media addiction was low and moderate. Unsurprisingly, the moderating effect of social media addiction did not occur when it was high. When they are highly engaged in social media, they may find an opportunity to meet their sense of belongingness with their peers in the virtual environment.
7	Zhen et al. (45)	The results demonstrated a complicated relationship between online self-disclosure and perceived stress. Specifically, we found a positive relationship between the levels of disclosing oneself to a small group of people and the levels of perceived stress. This positive relationship potentially indicates that college students are more likely to turn to their online friends through social media channels during stressful times. We also found that the willingness to disclose personal information to a selected group of people on social media moderates the negative impact of life disruptions such as schedule changes, moving, and selfisolating. However, the results did not show that peripheral selfdisclosure, such as updating SNS casually, helps relieve the stress.
8	Dong et al. (46)	In the present study, 2.68% and 33.37% of the participants were classified as addicted and possibly addicted to the Internet. The results also showed that IA grew with age. This study discovered that the frequency and duration of recreational electronic devices use, the frequency of electronic devices use after midnight, and the self-score of addiction to electronic products were all significantly higher than those before the epidemic in all the groups. Our data showed that a number of participants experienced significant depression, anxiety, and stress during the outbreak. In this particular period, due to the suspension of schools, the closure of living environments, the reduction of outdoor activities, and the increase of epidemic pressure, the mental health of school-age children and adolescents were threatened.
9	Vall-Roqué et al. (47)	The frequency of use of Instagram was positively associated with body dissatisfaction, drive for thinness and low self-esteem. However, effect sizes were very small, hence these results should be interpreted with caution. Following appearance-focused accounts on Instagram was found to be associated with drive for thinness in both age groups, and the effect size in this case was stronger, even though still small. Taking into consideration the lockdown's associated changes in SNS use stated above, these results might have significant implications, as they might indicate that the detrimental effects of SNS could have been exacerbated during the pandemic, and COVID-19 might be linked to increased drive for thinness and risk for eating disorder behaviors relative to media effects.
10	Sheoran et al. (48)	The total number of uncontrolled users of internet (moderately and severely addicted combined) constitute about two thirds (66.7%) of the sample population. There were, however, no significant gender differences in Internet Addiction among the male and female adolescents. In case of Psychological Distress, more than one third of the sample population (35.3%), was in moderate range and another 36.3 % population was found to be in the severe range. The female adolescents seem to be at a higher risk of Psychological Distress as compared to male adolescents though the differences with respect to gender are not statistically significant. The current study established significant positive correlation between Psychological Distress and Internet Addiction among adolescents.
11	Hong et al. (49)	EMS and covid info do not directly predict psychological distress. When college students read massive amounts of stressful news from various media platforms (e.g., WeChat), increased perceptions of COVID-19 as a severe health threat may lead to fear, anxiety, and depression. Thus, college students who are frequently exposed to COVID-19 information on social media are likely to report increased levels of anxiety and depression. Second, SME was positively associated with rumination, which in turn was positively associated with psychological distress in these college students, supporting the integrated model of ruminative response style. During this pandemic, exposure to COVID-19 information serves as a stressor, which may activate cognitive processes and increase ruminative thoughts. Mindfulness significantly moderated the first stage of the mediating process in these college students. Specifically, SME was positively associated with rumination among students who reported low levels of mindfulness, whereas this association was not significant among students who reported high levels of mindfulness.
12	Magson et al. (38)	The pre-pandemic to intra-pandemic increase in depressive symptoms and anxiety, and decrease in life satisfaction. The finding that girls are experiencing greater declines in mental health than boys during the COVID-19 crisis. The present results also showed that feeling socially disconnected during the pandemic was associated with higher levels of anxiety and depressive symptoms and lower levels of life satisfaction.
13	Li et al. (50)	Acute stress levels decreased over time while levels of possible depression and anxiety significantly increased. During second survey fewer participants said they use social media often and worry about their family or that they might get sick. Probability of developing symptoms related to acute stress increased in students with higher levels of depression and anxiety during initial spread of covid, higher exposure to social media, and anxiety about getting sick from covid.
14	Chambonniere et al. (51)	68.9% of adolescents reported an increased screen time. A higher proportion of children and adolescents who complied with the recommendations before lockdown reported an increase in their screen time (65.0% and 78.7% respectively) compared to those who exceeded the 2h/day of screen time before the lockdown (respectively 47.8% and 64.8%). A higher proportion of adolescents who had a sitting time > 6h/day before the lockdown declared an increase of their screen time during lockdown (70.3%) compared to those who showed a sitting time <6h/day before the lockdown (57.6%) (p < 0.001). All declared an increase in their screen time during the lock- down but the higher proportion of children and adolescents who lived in urban areas reported an increase in their time spent in front of screen (respectively 66.4% and 70.6%) compared 56.7% and 67.4% of those who lived in the countryside respectively. Higher proportions of children and adolescents who had not access to an outdoor before the lockdown admitted to increasing their screen time. A total of 64.2% of initially inactive adolescents before the lockdown reported an increase of their screen time during lockdown against 75.8% of the initially active participants (p < 0.001).
15	Islam et al. (52)	Problematic Internet use was significantly associated with being younger in age, having a bachelor degree level of education, being unmarried, being a member of a nuclear family, having middle-income socioeconomic status, living in an urban area, being a cigarette smoker, being a heavier sleeper, being physically inactive, not engaging in household chores, and having higher engagement with online activities (e.g., playing video games, social media use, and online recreational activities).
16	Parker et al. (53)	Among adolescents, just 7% met guidelines for moderate-to-vigorous physical activity (MVPA), which was slightly lower than the Australian average of 10% of 15- to 17-year-olds. Using platforms that promote physical activity determined that on average participants who used it added movement recommendations more often. 26.5% (255/963) of adolescents reported using digital platforms for physical activity. Adolescents'MVPA (OR 2.4, 95% CI 1.3-4.3), MSE (OR 3.1, 95% CI 2.1-4.4), and combined (OR 4.3, 95% CI 2.1-9.0) guideline adherence were also higher among users of digital platforms relative to nonusers.
17	Dragun et al. (54)	We found no substantial differences in dietary pattern between pre-lockdown and lockdown periods, including the overall Mediterranean diet (MD) adherence. MD adherence was positively correlated with QoL and study time, and negatively with TV and mobile phone use in pre-lockdown period (all p < 0.001). Interestingly, higher MD adherence was correlated with less perceived hardship and greater happiness and QoL during lockdown. The most prominent difference in sedentary activity corresponded to the time spent on computer/tablet. This kind of activity was reported to average three hours per day during COVID-19 lockdown, which is two hours more than before due to the online learning (p < 0.001). As many as 78.4% of secondary school students increased their computer time during lockdown period. subjective health rating was negatively correlated with daily sitting time, stress score, and anxiousness, while it showed a positive correlation with the MD adherence, sleep duration, quality of life, happiness and optimism during both study periods.
18	Fumagalli et al. (36)	Results showed that only social network usage increased in the initial stage of confinement as a function of lockdown initiation. Additionally, social network app usage was associated with increased feelings of loneliness, and this relation was mediated by fear of missing out (FOMO). In contrast, messaging app usage was associated with decreased feelings of loneliness, and was unrelated to FOMO. These results suggest that technology may be useful for mitigating the impact of loneliness during social isolation but that it is necessary to promote usage of messaging and VoIP apps, rather than social networking apps, because they are directly associated with decreases in loneliness without increasing FOMO.
19	Magis-Weinberg et al. (55)	Positive and negative online experiences were more frequent for older students, and females experienced more negative online experiences than males. Greater positive online experiences related to lower loneliness, with the reverse pattern for negative online experiences. Our results suggest that positive online experiences may mitigate loneliness during physical isolation. Lower loneliness was associated with lower negative experiences and also higher positive experiences.
20	Rens et al. (56)	The results indicate that about two thirds (65.49%) experienced mental distress. In the multivariable regression model, significant (p < 0.01) predictors of mental distress were female gender (OR = 1.78), low social support (OR = 2.17), loneliness (OR = 5.17), a small (OR = 1.63), or large (OR = 3.08) increase in social media use, a small (OR = 1.63) or large (OR = 2.17) decrease in going out for drinks or food, and a decrease in doing home activities (OR = 2.72).
21	Xiao et al. (57)	Physical activity, particularly of at least 150 minutes' duration each week, significantly decreased the likelihood of negative mood among adolescents during lockdown. Screen time, specifically other than that spent on online study, had a negative association with mood, after controlling for the relevant variables (i.e., physical activity and body mass index). Less screen time and accumulating 150 minutes of physical activity were associated with fewer conflicts with parents. An additional 1 hour of screen time that was not online study was associated with an increase of 1.6 to 1.8 points in participants' mood disturbance scores.
22	Nomura et al. (58)	Multivariable logistic regression analyses showed that risk factors for depression included being a woman, smoking, alcohol consumption, and social network communication using either video or voice. For suicide-related ideation, alcohol consumption was the only risk factor. Exercise and having someone to consult about worries were associated with decreased risk of both depressive symptoms and suicide-related ideation. Conclusions: Negative lifestyles of smoking and drinking, and being a woman, may be important risk factors for depressive symptoms, whereas exercise and having someone to consult about worries may be protective factors.
23	Hudimova et al. (59)	The study proves that young people spend almost all day online due to the obsessive pattern of social media involvement and/or procrastination, which often provokes withdrawal syndrome upon the attempt to distract from them. The lack of controlled time spending on social media during self-isolation provokes an exacerbation of anxiety, apathy, depressed mood, and a sense of isolation from social reality.
24	Cauberghe et al. (60)	Participants who were feeling lonely were more inclined to use social media to cope with lacking social contact. However, this coping strategy was not significantly related to their happiness feelings. Humorous coping was positively related with feelings of happiness, but not influenced by loneliness or anxiety. An analysis of the indirect effects showed that the effect of anxiety on happiness is positively mediated by social media (active) coping. A higher feeling of loneliness among the participants predicted social media use to keep in touch with peers and family, but it was not associated with happiness. Anxious participants indicated to use social media more often to actively seek for a manner to adapt to the current situation, and to a lesser extent as a way to keep in touch with friends and family. The indirect effect of anxiety on happiness through active coping was significantly positive
25	Pigaiani et al. (61)	When assessing the impact of adolescents' lifestyle behaviors and coping strategies on their psychological wellbeing, a number of variables predicted a significant change. “Active” and planning” adaptive coping strategies associated with a significant change in wellbeing included doing physical activity and engaging in different activities than before, including media use.
26	Islam et al. (52)	Problematic social media sue (PSMU) was positively associated with irregular physical exercise, poor engagement with academic studies, social media use, watching television, ignoring earning activities, anxiety, and depression. Similarly, PSMU was positively associated with lower age, poor sleep, alcohol consumption, social media use, anxiety, and depression. Moreover, according to the hierarchical regression analyses conducted, individuals with irregular physical activity were found to exhibit higher levels of PSPU than physically active individuals. The present study found that PSPU was significantly associated with poor study engagement. Moreover, the results of this study found that depression, and anxiety were positively associated with PSPU. In the hierarchical regression analysis conducted, reporting with less sleep (<7 h/day) were more prone to PSMU. Another important finding was that alcohol consumption and PSMU were positively associated.
27	Chen et al. (62)	In the present study, the problematic use of gaming, social media, and smartphones showed mediating effects in the associations between psychological distress (including depression, anxiety, and stress) and screen time use among Chinese primary schoolchildren during their school hiatus due to the COVID-19 outbreak. However, the fear of being infected by COVID-19 did not impact on any noticeable changes concerning increased time or problematic gaming, problematic social media use, and problematic smartphone use given the weak significant correlations with small effect sizes. In contrast, children who increased their time reading/studying or exercising showed less problematic use of Internet-related activities and less psychological distress during school hiatus.
28	Fung et al. (63)	We found that problematic smartphone use (PSU) was significantly higher during the COVID- 19 outbreak, However, there was no significant difference in PSMU across the three waves. we found positive associations between PSU/PSMU and psychological distress. However, their associations changed direction across the three waves. Specifically, the association between PSU and depression/anxiety decreased from before, during, to post-lockdown; however, association between PSMU and depression/anxiety increased from before, during, to post-lockdown. The diminished associations between PSU and depression/anxiety may be due to the recently designed mental health apps. Although we did not ask whether our participants used such apps, it could be possible that participants used apps to cope with their depression and anxiety. The exacerbated associations between PSMU and depression/anxiety may be due to the rumors and frightening news or information on COVID-19 in the social media.
29	Hayran et al. (64)	With two studies conducted at the beginning and towards the end of the pandemic, we tried to uncover the triggers and the accompanying well-being effects of university students' FOMO experiences resulting from this digital overuse. Our results reveal that during the pandemic, even when socially distancing at home, individuals continued to experience FOMO. During this time, there has been a major shift in the type and amount of digital information individuals consumed. Our findings show that FOMO has been commonly experienced due to the difficulty of catching up with real-time social media content, others' posts and videos, newly released movies and series on videostreaming platforms such asNetflix, and virtual gatheringswith family and friends. Especially individuals who are more prone to FOMO as a personality characteristic reported feeling it more intensely about digital content. We find that like a vicious cycle, higher involvement in virtual activities feeds into experiencing higher levels of FOMO, which then leads to increased engagement with social media.
30	Siste et al. (65)	This study observed a point prevalence of 19.3% for suspected Internet addiction among Indonesian adolescents during the COVID-19 outbreak. Our findings showed that internalization and externalization problems were correlated to higher KDAI scores. This finding corresponded with other studies about this issue on adolescents

In the following subchapters, the studies are summarized qualitatively and - when possible - quantitatively by grouping them according to the type of media use and its association with mental health, i.e. social media use and mental health, screen time (excluding social media use) and mental health, and media addiction and mental health. A separate subchapter focuses on longitudinal studies investigating the different types of media use and their causal relationships with mental health outcomes.

### Social Media Use and Mental Health During Covid-19

In general, studies reported that social media use increased during the Covid-19 pandemic (36, 47, 56, 60, 62), including the usage of a variety of social media platforms (e.g., Instagram, Snapchat, TikTok). In particular, three studies reported that about one-third of the participants used social media for more than 5 h per day (40, 50, 56), with some participants reporting time spent on social platforms up to 10 h per day (40).

**Figure 4:**
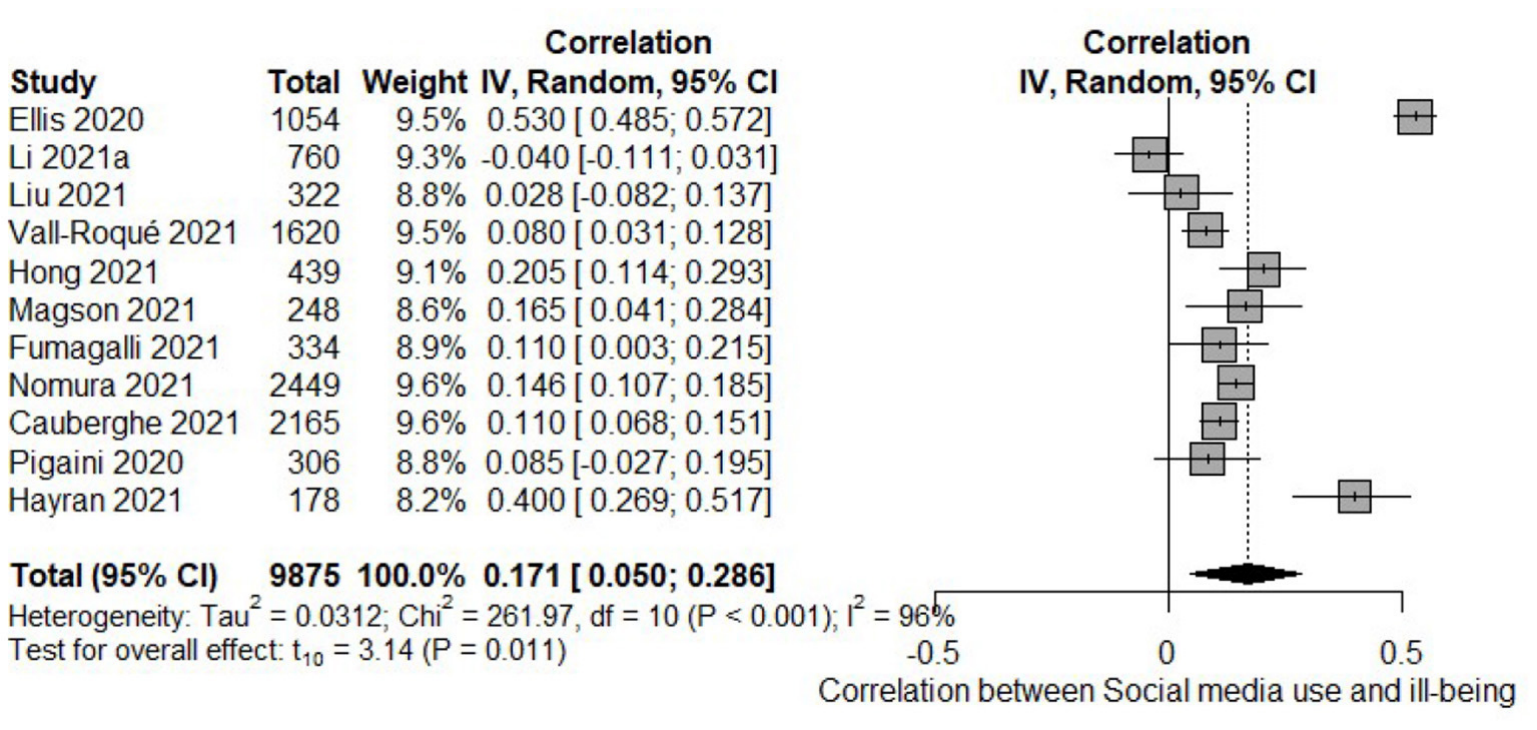
Forest plot of the meta-analysis of social media use and ill-being.

Overall, meta-analytic results showed that time spent on social media was positively correlated with ill-being (k = 11, *r* = 0.171, 95%CI [0.050–0.286], *p* = 0.011, I^2^ = 96%, Figure 4), indicating that using social platforms was related to higher psychopatological symptoms. To note, meta-regression analyses showed that the strength of correlation slightly augmented with age (β = 0.008, *p* = 0.027) and percentage of males in the sample (β = 0.333, *p* = 0.034). No significant association was found for measures of well-being (k = 6, *r* = −0.051, 95%CI [−0.194–0.0947], *p* = 0.411, I^2^ = 89%).

Looking at single studies examining ill-being, social media use was associated with higher levels of depression, anxiety, mental health problems in general, and lower self-esteem, especially among girls (38). Furthermore, girls reported having had negative online experiences more often (55). One study found that exposure to Covid-19 information likely increased levels of anxiety and depression, especially when participants had already reported psychopathological symptoms before the pandemic (50). Also, adolescents who - under normal circumstances - did not use social media so often reported a steeper increment in mental problems: Indeed, a three-fold increase in distress was reported in young people who augmented social media time up to 3 hours more than before the pandemic (56). Symptoms were also exacerbated through the mediating role of rumination - which refers to the persistent act of thinking about something bad, hurtful, or uncertain for an extended period (67). That was probably due to the greater exposure to Covid-19 related information online, although mindfulness skills buffered this adverse effect (49). A lack of cognitive control over the time spent on social media platforms likely exacerbated psychopathological symptoms and augmented a sense of isolation from social reality, fueling an obsessive cycle of social media usage patterns (59). Conversely, anxious participants reported using social media more often as a strategy to adapt to the current emergency and – to a lower extent – as a way to keep in touch with family and friends (60). Interestingly, individuals who reported more frequent experiences of Fear of Missing Out (FoMO) tended to use social media more frequently to seek and share information, thus fueling a vicious cycle and leading to an even higher engagement with these platforms (64). The higher engagement in social media content was carried out also if the information received were perceived as overwhelming (42). Furthermore, the use of Instagram and, in particular, following appearance-focused accounts was related to higher body dissatisfaction, drive for thinness and lower self-esteem in female adolescents. However, the effect sizes were small (47).

To note, conflicting results were found when social media use was considered in relation to social well-being (k = 5, *r* = −0.002, 95%CI [−0.181–0.176], *p* = 0.972, I^2^ = 94%), since studies reported both positive and negative associations. In particular, although depressive symptoms augmented – social media use mitigated the feeling of loneliness (40). However, the way through which this positive effect acted is complex. For example, disclosing oneself to a small group of people, i.e. reciprocal online friendship, was found to relieve stress more than one-to-many online communication (45). Similarly, a study found that messaging and the use of VoIP apps (e.g., Skype, Viber, WhatsApp) were associated with lower levels of loneliness. In contrast, general social media use increased feelings of loneliness via the mediating role of FoMO (36). A study of thirteen-year-old participants found that positive online experiences (e.g., feeling valued, receiving advice) decreased loneliness, whereas negative experiences (e.g., being cut-off and mistreated) augmented it (55). At the same time, one study showed that lonely participants were more inclined to use social media as a coping tool, but social media did not influence their happiness feelings (60). However, in the same study, humorous coping - e.g., watching or sharing funny videos - was positively related to feelings of happiness, although it was not influenced by loneliness or anxiety. These results underlined the positive associations between social media use and mental health in a developmental period during which connecting with peers is crucial for social well-being and showed that the *quality* and the *quantity* of social connections play a pivotal role.

Social media use was positively associated with Covid-19 related stress (k = 6, *r* = 0.253, 95%CI [0.049–0.437], *p* = 0.025, I^2^ = 93%). In particular, Covid-19 information shared through social platforms have been perceived as excessively complex and overwhelming, thus augmenting both social media fatigue and fear of Covid-19 (42), with the risk to further bias information-processing capacities for the assessment of Covid-19 information. Conversely, young people reporting more Covid-19-related stress fostered active use of social media as a coping tool [e.g., (40, 43)].

Lifestyle behaviors closely linked to mental health were also associated with time spent on social media. More precisely, higher levels of social media use were associated with lower levels of physical activity, more frequent sleep problems, as well as higher levels of substance use. In the meta-analysis, including two studies (40, 41), a non-significant relationship between lifestyle behaviors and social media use during the pandemic was found.

### Screen Time and Mental Health During Covid-19

Screen time included any screen-based media use except for video gaming and social media use (excluded, unless studied alongside other screen-based activities). The majority of the included studies looking at screen time found that it augmented during the pandemic, especially for online leisure activities, watching television, news consumption, and overall Internet usage through smartphones, computers, and tablets (37, 46, 50, 51, 54, 62, 65). In one study, participants reported spending up to 11 h and more per day in front of screens (65). Also, young people living in urban areas reported an additional increase in their time spent in front of screens compared to those living in the countryside (51).

Two studies reported comparable effect sizes for screen time and well-being, the latter measured as general well-being and happiness (41, 54). The meta-analytic results revealed a negative yet marginally significant pooled correlation (k = 2, *r* = −0.196, 95%CI [−0.429–0.061], *p* = 0.065). The same studies also reported comparable effect sizes on screen time and ill-being. However, meta-analytic results were not significant.

Looking at the individual studies, the frequency and duration of recreational screen media use, as well as nighttime use, augmented, and this increment was related to increased psychopathology (46). Particularly, leisure screen time was negatively associated with mood problems, even after considering covariates such as physical activity and body mass index (57). In addition, young people showed less increment in Internet-related activities and lower psychological distress when involved in structured activities (37) or spent more time reading, studying or exercising (62).

Screen time that was not used for social interactions was negatively related to social well-being (k = 2, *r* = −0.115, 95%CI [−0.178–−0.051], *p* = 0.028). Meta-analytic results linking screen time and lifestyle behaviors were not significant. However, increased sitting and screen time was followed by a precipitous decline in physical activity, which led to lower mood levels (57). To note, the increment in screen and sedentary time was reported irrespectively of the initial time dedicated to both activities (51). Participants with previous mental health problems were also at higher risk of an unhealthy lifestyle, including lower levels of physical activity, higher levels of screen time, and poorer sleep quality (41). On the contrary, when adolescents used digital platforms promoting physical activities, they were more likely to meet the recommended movement guidelines (53). Also, lower television and mobile phone use levels were related to greater adherence to a Mediterranean diet, which was, in turn, related to less perceived adversity and more happiness and quality of life during the lockdown (54).

Finally, although a meta-analysis was not possible due to the paucity of studies, participants reported that increased screen time, including news consumption, helped them stay up-to-date and cope with Covid-19 uncertainty, although news consumption also augmented fear of infection (37).

### Media Addiction and Mental Health During Covid-19

Across the studies included in this review, prevalence rates of media addiction ranged from about 20 to 70% (44, 48, 52, 63, 65). Two studies revealed that media addiction levels grew during the pandemic (46, 48). A study found that media addiction was more prevalent among youth who had difficulties organizing their daily schedules (59).

**Figure 5:**
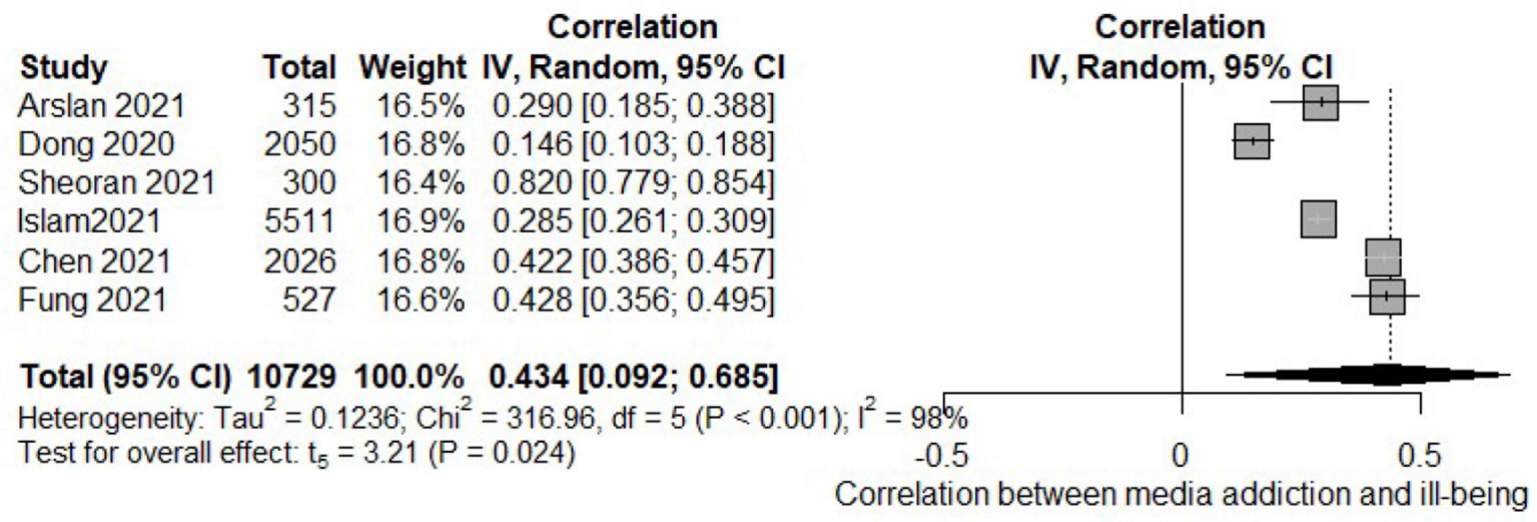
Forest plot of the meta-analysis of media addiction and ill-being.

During the Covid-19 pandemic, media addiction showed a medium-to-large positive relation to ill-being (k = 6, *r* = 0.434. 95% CI [0.092–0.685], *p* = 0.024, I^2^ = 98%, Figure 5) including internalizing and externalizing problems [e.g., (65)]. Looking at the different types of addiction, Fung et al. (63) found a positive association between social media addiction and ill-being, likely caused by rumors and alarming news on Covid-19 circulating on social media platforms. Nevertheless, the same authors reported a decrease in the strength of the association between depression and anxiety with smartphone addiction over time, possibly due to the recently designed mental health apps (63). Also, according to Siste et al. (65), adolescents were more susceptible to Internet addiction than young adults during Covid-19. The authors explained these findings with the fact that adolescents' cognitive control system is still underdeveloped. On top of that, the pandemic has limited physical peer contacts essential for adolescents' growth and social connection, pushing adolescents to alternative, online means to stay in contact with peers and friends. Media addiction also deteriorated psychological adjustment via college belongingness: Students with higher levels of college belongingness reported better psychological adjustment. However, when social media addiction was high, it likely interfered with the sense of belonging to the school (44).

Concerning the association between media addiction and lifestyle behaviors, the former was related to irregular physical exercise or physical inactivity, lower engagement with studying, ignoring earning activities and household chores, poor or heavy sleep, and alcohol or cigarettes consumption (52, 66). Yet, the meta-analytic results based on the comparable effect sizes of the two studies were not significant.

### Longitudinal Studies

Six studies included in this review used a longitudinal design (36–38, 50, 55, 63), thus providing insights into the causal mechanisms between (addictive) media use and mental health. In particular, using Ecological Momentary Assessments (EMAs) for 14 days, Arend et al. (37) found that more than 40% of participants augmented daily time spent watching TV and using social media. Participants who reported frequent experiences of structured daily activities also engaged in less intense sessions of screen-based activities, like video gaming, Internet surfing, and television watching. Fumagalli et al. (36) obtained screen-time usage data for a 4-week period from diverse countries. They found that only social media use augmented at the beginning of the lockdown in spring 2020. Furthermore, higher levels of social media use predicted higher levels of loneliness through the mediating role of FoMO. On the contrary, messaging and VoIP apps usage reduced loneliness and was not influenced by individuals' FoMO levels. Also, VoIP apps consumption varied greatly among countries, but time spent using these apps was still lower with respect to time spent using social media apps. Magis-Weinberg et al. (55) studied levels of loneliness and reported that they remained unchanged between weeks 6 and 11 of the lockdown in Spring 2020 in Perù. Yet, loneliness was consistently more prevalent among females. Social media use, including positive experiences, such as feeling valued and receiving advice, predicted lower levels of loneliness over three months, whereas negative experiences on social media as well as overall screen time predicted the opposite. Furthermore, one study (38) reported an increment in depressive and anxiety symptoms and a decrease in life satisfaction from the pre-pandemic to the intra-pandemic period. However, exposure to Covid-19 information on social media did not significantly affect these changes in mental health. Fung et al. (63) collected data from 11-years-old participants during the pre-, ongoing-, and post- Covid-19 lockdown, finding that the positive association between smartphone addiction and ill-being decreased across the three waves. The opposite happened for social media addiction, for which the association with depression and anxiety increased across the three waves. Finally, the study by Li et al. (50), including 1,64,101 Chinese college students at the onset of the Covid-19 in February 2020 and 68,685 participants at the follow-up assessment - about 2.5 months later - highlighted that acute stress diminished. However, depressive and anxiety symptoms augmented, and social media exposure was a risk factor, especially when participants spent more than 3 h per day with these platforms.

## Discussion

During the Covid-19 pandemic, especially during the lockdown, social distancing measures and the associated disruption of everyday activities and social contacts threatened the mental health of adolescents (6, 68). To alleviate the negative experiences of social distancing measures, young people spent more time online. Lockdown and distancing measures began not long ago, but researchers have studied whether and how the time spent in front of screens affected mental health. However, a comprehensive synthesis of the literature published so far was still missing. Based on a systematic search and screening process, the present review qualitatively and quantitively summarized the existing evidence on the association between (addictive) screen media use and mental health in adolescents during the Covid-19 pandemic.

The key message of the present review is that *not all uses of digital media had negative consequences* on adolescents' mental health during the pandemic. In particular, our results suggested that social media use was helpful in mitigating the feeling of loneliness during Covid-19, but only when a one-to-one or one-to-few communication (e.g., use of VoIP apps), rather than a general social media use, was promoted. Likewise, online disclosure in the context of reciprocal friendship was found to relieve stress rather than a one-to-all peripheral disclosure on social media. In addition, good online experiences like receiving positive feedback augmented social connection and reduced loneliness during the lockdown, and using social media as a humorous coping tool (i.e., using humor to cope with the pandemic) increased happiness. These findings align with the Theory of Compensatory Internet Use, according to which “negative life situations can give rise to a motivation to go online to alleviate negative feelings” [(69), p. 352], although time online can have both positive and negative outcomes. In the present review, positive outcomes included better self-reported (social) well-being and less ill-being in terms of loneliness and stress.

Although some studies underlined the positive side of social media use, the majority reported that digital media use was associated with diminished well-being. Firstly, detrimental effects of social media may derive from the overload of Covid-19-related information, which was frequently negatively valenced and included much misinformation augmenting feelings of worry, fear of the pandemic, and FoMO, thus diminishing well-being [e.g., (42)]. It is likely that, when dealing with high levels of stress and uncertainty, online communication and posts' sharing among adolescents fostered rumination on negative feelings and involuntarily intensified these concerns. Not surprisingly, older participants, who better understood the pandemic's severity, were more affected by the negative consequences of social media contents' exposure.

Secondly, young people used social media as a coping tool to disconnect from negativity, avoid boredom, displace time for homework, get entertained and follow social media content without getting directly involved. However, as reported by previous literature reviews, passive and compensatory social media use led to increased ill-being (59), including feelings of depression, anxiety, loneliness, and low self-esteem due to social comparison as well as body-related concerns (47). The latter result is in line with a general risk of increased eating disorders during the pandemic (70). Although many of the studies included in this review used a correlational design, the results indicated a detrimental effect of social media use during the Covid-19 pandemic, especially in adolescents who were less involved in online activities and those who already experienced mental health problems before the pandemic.

While mechanisms such as social comparison, FoMO or exposure to negative Covid-19-related information are one possible explanation for the detrimental effect of social media, another mechanism is that social media, and screen time in general, replaced lifestyle activities promoting mental health. For example, more time in front of screens was associated with sleep problems, leading to even more screen time during night hours and determining irritability and anxiety. Likewise, physical activity was reduced, and a more sedentary lifestyle was adopted, including a poorer diet and greater use of substances like alcohol and cigarettes, which were subsequently associated with more screen time [e.g. (54)] and a more frequent engagement in alcohol-related social media usage [e.g., (66)].

Additionally, youth who spent more time in front of screens to deal with Covid-19 stressful situations also tended to fall into “an immersed pleasant state through repeated use”, which eventually led to the development of media addiction symptoms (43). Yet, the prevalence of these addictive symptoms varied considerably across the included studies, ranging from 20 to 70 per cent. In particular, during home quarantine, social media use was the only way to meet and socialize, thus contributing to longer time spent online and more frequently reported symptoms of social media addiction, especially when adolescents experienced FoMO, thus diminishing well-being (64) - in particular, FoMO proneness was higher at the early stage of the pandemic (64). Given that adolescents' self-control skills are still underdeveloped due to the immaturity of the prefrontal cortex, younger age groups were even more at risk of developing symptoms of media addiction during the pandemic, which was further facilitated by the instantaneous and easy-to-access gratifying contents that (social) media platforms convey (65, 71). According to the Interaction of Person-Affect-Cognition-Execution Model (72), the experience of psychological distress, such as the one created by the Covid-19 lockdown, likely contributed to the development of addictive Internet use. As previously stated, social media, and screen media in general, could be used as a coping tool for self-regulating negative emotions deriving from stress, fear, uncertainty, and lifestyle changes due to the pandemic. That would end up in the experience of negative emotions due to the loss of control over online activities and the search for more frequent gratifications online (62). This would explain the sizeable meta-analytic correlation found between media addiction measures and ill-being.

To conclude, although overall digital media use was related to lower adolescents' well-being during Covid-19, some kind of social media use (i.e., one-to-one communication and online mutual relationships, the experience of funny and positive contents) improved social and mental well-being and helped adolescents to deal with the lack of in-person social experiences during the pandemic. That said, our findings contribute to a growing body of evidence highlighting that the *quality* rather than the *quantity* of online interactions and experiences are crucial in determining potential influences on young people's mental health. Thus, the positive aspects of online activities should be promoted. At the same time, awareness should be raised about the detrimental effects of addictive media use and adverse mechanisms such as social comparison, FoMO, and the exposure to negative content during online activities, which can happen more frequently in times of pandemic, social isolation, and confinement.

## Limitations and Future Directions

This review does not come without limitations. We included only peer-reviewed articles; hence, pre-prints and gray literature were left out, which may have introduced some biases. Also, in some cases, we did not have enough effect sizes to conduct a meta-analysis on all of the associations of interest or to run meta-regression and sub-group analyses. Additionally, heterogeneity levels of the effect sizes varied largely, suggesting a significant variance that other factors should likely explain. Also, more studies are needed to conclude on more reliable results. Eventually, the findings of our systematic review may be biased because the included studies looked mainly at detrimental effects of screen time and social media use, including addictive use, with only a few focusing on a positive conceptualization of mental well-being. Likewise, causality claims, i.e., whether screen time and social media use impact mental health or whether the latter is a driver of certain usage behaviors, could only be made with caution since most reviewed studies relied on a cross-sectional design.

We encourage researchers to focus on the positive side of mental health for future research, including *hedonic* and *eudaimonic* well-being measures. Furthermore, given the strong focus and predominance of studies measuring the *quantity* of (social) media use in terms of duration and frequency, future studies should move beyond these holistic measures and disentangle the type and *quality* of (social) media use. Furthermore, researchers should invest time and effort in longitudinal studies, although we are confident that more longitudinal findings will be published in the upcoming months. Researchers should include longer time ranges when conducting longitudinal studies and use a rigorous statistical procedure to differentiate between- and between-person effects.

## Conclusions

To conclude, the present systematic review and meta-analysis first summarizes the existing evidence from 30 studies on the link between mental health and digital media use in adolescents during Covid-19. Results showed that adolescents augmented their social media use, including general screen time. Also, higher levels of digital media addiction were reported during the pandemic. In general, higher social media use and media addiction were related to higher ill-being. Hence, adolescents are particularly at risk of experiencing mental health problems due to the augmented exposure to screen time and social media during the pandemic. However, not all types of digital media use had a negative consequence. In particular, one-to-one communication, mutual online friendship, and positive and funny online experiences mitigated feelings of loneliness and stress during Covid-19. These positive aspects of online activities should be promoted. Youth's access to psychological support services to provide measures for and promote healthy coping mechanisms during the ongoing Covid-19 pandemic should be facilitated (68).

## Data Availability Statement

The original contributions presented in the study are included in the article/supplementary material, further inquiries can be directed to the corresponding author/s.

## Author Contributions

LM and MO contributed to developing the main research question, carrying out the literature search, collecting the included studies' information, and describing the results. LM performed the meta-analysis and wrote the first draft of the manuscript. A-LC contributed to developing the main research question and revised the manuscript. PS revised the manuscript. All authors contributed to the article and approved the submitted version.

## Funding

This research was in part funded by the Swiss National Science Foundation (Grant No. 175874).

## Conflict of Interest

The authors declare that the research was conducted in the absence of any commercial or financial relationships that could be construed as a potential conflict of interest.

## Publisher's Note

All claims expressed in this article are solely those of the authors and do not necessarily represent those of their affiliated organizations, or those of the publisher, the editors and the reviewers. Any product that may be evaluated in this article, or claim that may be made by its manufacturer, is not guaranteed or endorsed by the publisher.
